# Health-related articles on Syria before and after the start of armed conflict: a scoping review for *The Lancet*-American University of Beirut Commission on Syria

**DOI:** 10.1186/s13031-020-00316-7

**Published:** 2020-11-05

**Authors:** Rima A. Abdul-Khalek, Walaa Kayyal, Abdul Rahman Akkawi, Mohamad Almalla, Khurram Arif, Lama Bou-Karroum, Amena El-Harakeh, Manal K. Elzalabany, Racha Fadlallah, Fatima Ghaddar, Danna Kashlan, Sara Kassas, Tania Khater, Nisreen Mobayed, Dalal Rahme, Omran Saifi, Samer Jabbour, Fadi El-Jardali, Elie A. Akl, Mohammed Jawad

**Affiliations:** 1 Faculty of Health Sciences, American University of Beirut, Beirut, Lebanon; 2 Faculty of Medicine, American University of Beirut, Beirut, Lebanon; 3 Center for Systematic Reviews on Health Policy and Systems Research (SPARK), American University of Beirut, Beirut, Lebanon; 4 Medical College of Wisconsin, Milwaukee, WI USA; 5 AUB Libraries, American University of Beirut, Beirut, Lebanon; 6 Clinical Research Institute, American University of Beirut Medical Center, Beirut, Lebanon; 7 Public Health Policy Evaluation Unit, Imperial College London, London, UK

**Keywords:** Public health, Syria, Research, Productivity, Health-related articles, Conflict

## Abstract

**Introduction:**

Armed conflict may influence the size and scope of research in Arab countries. We aimed to assess the impact of the 2011 Syrian conflict on health articles about Syria published in indexed journals.

**Methods:**

We conducted a scoping review on Syrian health-related articles using seven electronic databases. We included clinical, biomedical, public health, or health system topics published between 1991 and 2017. We excluded animal studies and studies conducted on Syrian refugees. We used descriptive and social network analyses to assess the differences in rates, types, topics of articles, and authorship before and after 2011, the start of the Syrian conflict.

**Results:**

Of 1138 articles, 826 (72.6%) were published after 2011. Articles published after 2011 were less likely to be primary research; had a greater proportion reporting on mental health (4.6% vs. 10.0%), accidents and injuries (2.3% vs. 18.8%), and conflict and health (1.7% vs. 7.8%) (all *p* < 0.05); and a lower proportion reporting on child and maternal health (8.1 to 3.6%, *p* = 0.019). The proportion of research articles reporting no funding increased from 1.1 to 14.6% (*p* < 0.01). While international collaborations increased over time, the number of articles with no authors affiliated to Syrian institutions overtook those with at least one author affiliation to a Syrian institution for the first time in 2015.

**Conclusion:**

To our knowledge, this is the first study to examine the impact of armed conflict on health scholarship in Syria. The Syrian conflict was associated with a change in the rates, types, and topics of the health-related articles, and authors’ affiliations. Our findings have implications for the prioritization of research funding, development of inclusive research collaborations, and promoting the ethics of conducting research in complex humanitarian settings.

**Supplementary Information:**

**Supplementary information** accompanies this paper at 10.1186/s13031-020-00316-7.

## Background

Research productivity in Arab countries is estimated to be lower than the international average [1–3]. One bibliometric analysis showed that Arab countries produced fewer biomedical publications than other Middle Eastern countries between 2001 and 2005, even after adjustment for population size and gross domestic product (GDP) [4]. These journal publications were also of lower quality when measured through the h-index and impact factor criteria. Such a pattern is evident in bibliometric analyses using different indicators such as the number of citations per publication and the h -index [5]. Between 2007 and 2016, Arab countries lagged behind other countries globally, producing 189 medical research papers per million population compared with 695 paper per million population for other world countries [1].

While studies have shown that Arab countries have generally scored low when assessing peer-reviewed publication rate, a Medline search for the 1996–2012-time period identified Syria as having the lowest number of journal publications among countries of the Eastern Mediterranean Region (EMR), i.e. 0.88 per 100,000 population [6]. In terms of research and development expenditure (% GDP), Syria had the second lowest reported expenditure of 0.02053 for the year 2015 [7]. This indicator is low or unreported in neighboring countries and is compared to 0.46223 for the Middle east and North Africa and 2.093 for the world average.

In Syria, anti-government protests have escalated to an armed conflict, ongoing since 2011, and have resulted in one of the largest humanitarian crises in recent eras. The crisis has had profound impacts on different industry sectors and on the health of civilians remaining inside Syria. It has also attracted donors and researchers to Syria, resulting in increased interest in the health and livelihood of Syrians. Generally, the effect of armed conflict is expected to extend to research productivity; in fact, armed conflict has been suggested as one of the explanations for a lower research productivity in Arab countries compared to other countries [8]. A recent bibliometric analysis found that countries experiencing armed conflict had a lower number of research publications compared to more stable ones [9]. The determinants of a countries’ research productivity (including quantity, quality and type) include the GDP and government health expenditure [10]; two factors that are severely hampered in times of armed conflict.

While conflicts affect research productivity negatively when examined through the lens of institutional productivity, knowledge production about conflict-affected countries and research collaborations may be affected differently. Assessing the impact of research productivity in conflict affected countries by estimating international research on the affected country, as well institutional research in the same country, can give a more complete picture on research productivity in conflict. The Ivory Coast for example, a country affected by a civil war, experienced an increase in research productivity, with a drop in Ivorian first or last authors in journal publications and the internationalization of the Ivory Coast’s research productivity [11]. In the EMR, one study has looked into the relationship between different forms of political instability and scientific research in the Arab world using one database [9]. Most countries in the Arab Spring had an increase in research productivity measured by institutional affiliations, but were mostly experiencing protests, rather than armed conflicts. To our knowledge, no study has assessed the effect of armed conflict on research productivity and international collaborations for health-related publications on Syria. Therefore, in this study, we aimed to assess the impact of the 2011 Syrian conflict on published journal health articles on Syria, comparing pre-2011 to post-2011 research productivity.

## Methods

### Study design

We conducted a bibliometric and content analysis of published articles, following the PRISMA Extension Checklist for reporting scoping reviews and used a protocol for data collection and analysis.

### Search strategy

In April 2017, we searched the following seven electronic databases without language or study design restrictions: Embase, Global Health Library, Medline, PsychInfo, PubMed, Scopus, and Web of Science. For each database, we used a combination of MeSH and free-text keywords related to Syria and health (full search strategy Table S1, Additional file 1). We restricted articles to those published after 1990, which is considered the start of economic liberalization reforms in Syria [12]. Our approach of restricting to articles published after 1990 is supported by the findings of a bibliometric review that showed only a small number of research articles from the Arab region were published in the early 1990s [13].

### Eligibility criteria

We included articles if they were about Syria, about clinical, biomedical, public health, or health systems topics, and were published after 1990. We included articles about Syria regardless of authors’ country of affiliation in order to capture all articles about Syria. We excluded articles conducted on animals or plants. Because one of our objectives was to assess changes in authorship and collaborations with Syrian institutions following armed conflict, we excluded articles on Syrian refugees, as these do not necessarily include collaborations with Syrian institutions.

### Selection process

We screened the titles and abstracts of retrieved citations independently and in duplicate, including articles judged as potentially eligible by at least one reviewer. We then conducted full text screening, independently and in duplicate, and resolved disagreements by discussion or by consulting a third reviewer. We excluded articles when a full-text version was not available, when the article was not related to health or was not about Syria, or when we were unable to obtain a translation.

### Data abstraction

Two reviewers (MJ and RAA) used a standardized data abstraction form to collect and manage bibliometric and content data. They conducted calibration exercises beforehand to ensure the consistency of data abstraction. Collected data included the year of publication, number of authors, presence of any Syrian author, country of affiliation of the first, senior and corresponding author(s) (and for Syrian affiliations, the name of the first-listed institution), type of publication (e.g. new, primary research, etc.), study design, name, and journal name. If an author’s country of affiliation was not reported, we searched for same-year publications by that author and used the reported country of affiliation if found to be consistent. In the absence of same-year publications, we expanded the search to two years on either side of the publication year and used the reported country of affiliation if found to be consistent. In cases of inconsistency, we conducted online searches (e.g. ORCID, LinkedIn, staff institutional websites) to determine the author’s affiliation at the time of the study. For primary research articles, we abstracted information on the reporting of funding and funding source. For public health primary research studies only (which include the involvement of research participants) we abstracted data on obtaining ethical approval.

We used the World Health Organization (WHO) categorization of health areas to assign articles to one or more of the three categories: public health, health systems and clinical/biomedical. Topic areas in public health included mental health, communicable diseases, non-communicable diseases, dental health, accidents and injuries etc.). We used the WHO building blocks framework to classify publications as belonging to one or more of the following six health system areas: service delivery, health workforce, health information systems, essential medicine, financing, and leadership governance [14].

### Data analyses and data visualization

We categorized publications into pre- and in-conflict time periods based on the year of publication. Articles published before 2011 were considered pre-conflict, and articles published after 2011 were considered in-conflict. Articles published in 2011 were considered pre-conflict for original research (given the known lag time from data collection to publication), and in-conflict for news, letters, editorials, and reports.

We summarized categorical variables using frequencies and percentages. We then calculated the pre- to in-conflict percentage change in the frequency of articles per country of affiliation of the first author. We also compared the yearly change in the presence of any Syrian author using non-parametric regression scatterplot with Epanechnikov kernel and 0 degree. We ranked journals by the frequency of articles (for journals with a minimum of 20 articles). We also assessed trends in number of published articles by comparing frequencies and percentages across years. For the trend analysis we only included articles published until 2016 to ensure we had a complete year of analysis (we conducted our search part of way through 2017). Pre- and in-conflict periods were compared using Chi2 and Fisher’s exact tests at an alpha level of 0.05. All statistical analyses were conducted using STATA v.15.1 software [15].

We conducted a social network analysis of author affiliation using the Gephi tool v.0.9.2 and the Forced Atlas algorithm for the visualization of results [16]. In the social network analysis graphs, each node represented the country of affiliation of the author. The network reflected the connectivity between the affiliation of the first author and the affiliation of the senior author in each article, and whenever the first and senior author were from the same country, a self-loop was created. We measured the “degree centrality”, indicated by the size of the node, to assess which node had more links with other nodes, i.e. more connectivity between countries. The thickness of the edge indicated the frequency of the collaboration between two nodes. The nodes were color-coded by modularity, which is the fraction of the edges that fall within the given groups minus the expected fraction if edges were distributed at random. Modularity was used to measure the structure of the network, and the strength of division of the network to clusters or communities. We restricted this analysis to articles with more than one author, to allow network analysis, and where the country of affiliation of authors was reported.

## Results

### Study flow

**Fig. 1:**
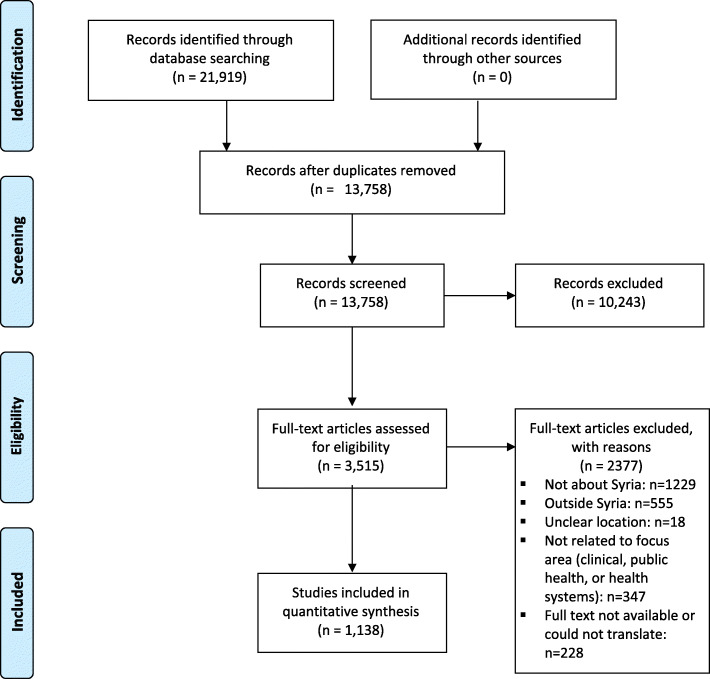
Study flow chart

Out of 21,919 articles retrieved by our search, 3515 were eligible for full-text screening, and 1138 met the inclusion criteria and were included in our analysis (Fig. 1). The main reason for excluding articles was because their focus was about countries other than Syria (*n* = 1784).

### Yearly trends

**Fig. 2:**
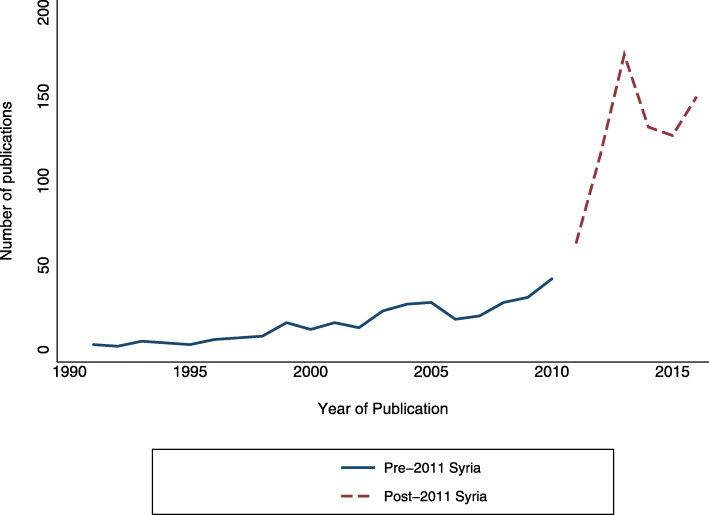
Publications on Syria before and after the start of the Syrian conflict

Of 1138 articles, 312 (27.4%) were pre-conflict and 826 (72.6%) were in-conflict. The number of articles slowly increased between 1991 and 2010 (from 3 to 42), only to rise sharply by over 300% between 2010 to 2013 (42 to 176) and plateau at 150 articles in 2016 (Fig. 2).

### Article type and topic

The journals that published most of the pre-conflict Syrian health articles were the *Saudi Medical Journal* (7.1% of all articles) and *Eastern Mediterranean Health Journal* (6.4%); during in-conflict period, these journals were replaced by *The Lancet* (7.8%) and *British Medical Journal* (4.0%) (Additional file 1, Table S2). The change in journals coincided with increased publication of news articles (1.9% vs. 7.9%), editorials/commentaries (6.0% vs. 16.3%) and secondary research (9.9% vs. 11.3%), and decreased publication in primary research articles (67.9% vs. 49.2%) (all *p* < 0.01) (Table 1).

**Table 1:** Type of articles before and after the 2011 Syrian conflict, n (%)

		Total (***N*** = 1138) n%	Pre-conflict (***N*** = 312) n%	In-conflict (***N*** = 826) n%	***p***-value^**a**^
Type of article	News	72 (6.3)	6 (1.9)	66 (7.9)	< 0.001
	Editorials, commentaries, opinion pieces	154 (13.5)	19 (6.0)	135 (16.3)	
	Primary research	619 (54.4)	212 (67.9)	407 (49.2)	
	Secondary research	125 (11.0)	31 (9.9)	94 (11.3)	
	Letter to editor	53 (4.7)	6 (1.9)	47 (5.6)	
	Research letter	3 (0.3)	1 (0.3)	2 (0.2)	
	Report	29 (2.6)	8 (2.5)	21 (2.5)	
	Conference proceeding	65 (5.7)	25 (8.0)	40 (4.8)	
	Other	18 (1.6)	4 (1.2)	14 (1.6)	

^a^Chi-squared difference in proportions

Between pre- and in-conflict periods, no relative change was found for articles broadly categorized as public health, clinical, or biomedical, but an increased proportion of articles published about health systems was evident (10.2% vs. 24.2%, *p* < 0.001) (Table 2). Broken down by WHO building blocks framework for health systems, no significant changes in health system topic areas were found (Table 2). For articles broadly about public health, in-conflict compared to pre-confict articles had a greater proportion reporting on mental health (4.6% vs. 10.4%; *p* = 0.029), accidents and injuries (2.3% vs. 18.7%, *p* < 0.001), and conflict and health (1.7% vs. 7.8%, *p* = 0.004), and a lower proportion reporting on child and maternal health (8.0% vs. 3.5%, *p* = 0.019), and sexual and reproductive health (5.7% vs. 1.1%, *p* = 0.001) (Table 2).

**Table 2:** Topic area of publications (public health and health systems only) before and after the start of the 2011 Syrian conflict, n (%)

	Total (***N*** = 1138) n%	Pre-conflict (***N*** = 312) n%	In-conflict (***N*** = 826) n%	***p***-value
Public Health	622 (54.6)	174 (55.7)	448 (54.2)	0.643
Health Systems	232 (20.3)	32 (10.2)	200 (24.2)	< 0.001
Clinical/Biomedical	391 (34.3)	116 (37.1)	275 (33.2)	0.218
**Public Health**				
Mental health	53 (8.5)	8 (4.6)	45 (10.4)	0.029
Non-communicable disease	135 (21.7)	35 (11.2)	100 (12.1)	0.679
Communicable disease	160 (25.7)	42 (13.4)	118 (14.2)	0.721
Dental Health	35 (5.6)	11 (3.5)	24 (2.9)	0.589
Environmental health^a^	10 (1.6)	2 (0.6)	8 (0.9)	0.734
Nutrition	37 (5.9)	11 (6.3)	26 (5.8)	0.806
Accidents and injuries	88 (14.1)	4 (2.3)	84 (18.7)	< 0.001
Conflict and Health	38 (6.1)	3 (1.7)	35 (7.8)	0.004
Genetic^a^	8 (1.2)	2 (1.1)	6 (1.3)	1.00
Human Rights^a^	17 (2.7)	2 (1.1)	15 (3.3)	0.174
Child and Maternal Health	30 (4.8)	14 (8.0)	16 (3.5)	0.019
Sexual and Reproductive Health^a^	^a^15 (2.4)	10 (5.7)	5 (1.1)	0.002
Quality of life^a^	^a^2 (0.4)	1 (0.5)	1 (0.2)	0.482
Tobacco	48 (7.7)	33 (18.9)	15 (3.3)	< 0.001
**Health System**				
Service delivery	120 (51.7)	13 (40.6)	107 (53.5)	0.176
Health workforce	106 (45.6)	13 (40.6)	93 (46.5)	0.536
Health informatics^a^	6 (2.5)	1 (3.1)	5 (2.5)	0.594
Healthcare access^a^	24 (10.3)	1 (3.1)	23 (11.5)	0.214
Financing^a^	16 (6.9)	2 (6.2)	14 (7.0)	1.00
Leadership/Governance^a^	13 (5.6)	1 (3.1)	12 (6.0)	1.00

Chi squared test and ^a^Fisher's exact test; Articles may fall into more than one topic area, public health or health system area

### Funding and ethical approval

Between pre- and in-conflict periods, the percentage of research articles reporting funding decreased from 29.8 to 25.8%, while there was an increase in reporting of no funding (1.1 to 14.6% (Table S3, Additional file 1). Among all articles that reported funding (*n* = 231), the most commonly reported funders were Damascus University (12.1%), United States Public Health Service (USPHS) (11.6%), and the Atomic Energy Commission of Syria (10.3%). In-conflict period funders that were not reported in the pre-conflict period included the Atomic Energy Commission of Syria (*n* = 24), University of Aleppo (*n* = 8), and the Bill and Melinda Gates Foundation (*n* = 4) (Table S4, Additional file 1).

Between pre- and in-conflict periods, specifically for primary research articles in the area of public health or health system involving a study population (*n* = 234), there was an increase in the percentage of articles reporting ethical approval (27.8 to 61.0%, *p* = 0.001).

### Syrian authors and institutions

**Fig. 3:**
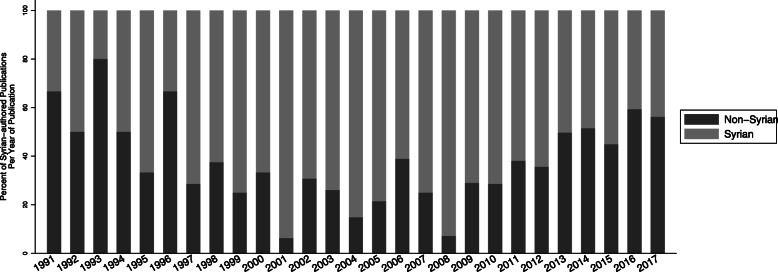
Trends in Syrian vs. non-Syrian institutional affiliations of authors publishing on Syria

Between pre- and in-conflict periods, the percentage change in articles with no authors affiliated to a Syrian institution increased from 28.5 to 59.3% (Fig. 3). A regression analysis showed that the number of articles with no authors affiliated to Syrian institutions overtook those with at least one author affiliation to a Syrian institution in 2015 (Figure S1, Additional file 1). The total number of articles with at least one author affiliated to a Syrian institution was 653 (57.4%). Pre-conflict, 229, i.e. 73.4% of all pre-conflict articles had at least one author affiliated to a Syrian institution. In-conflict, although the number increased to 424 articles, it represents 51.33% of articles published during that period (Fig. 3 and Figure S1). Among articles with at least one author affiliated to a Syrian institution, the most common institutions of affiliation were Damascus University (47 (20.4%) vs 148 (34.7%)), University of Aleppo (15 (6.5%) vs 30 (7.0%)) and the Syrian Center for Tobacco Studies (28 (12.1%) vs 16 (3.7%)).

### International collaborations

**Fig. 4:**
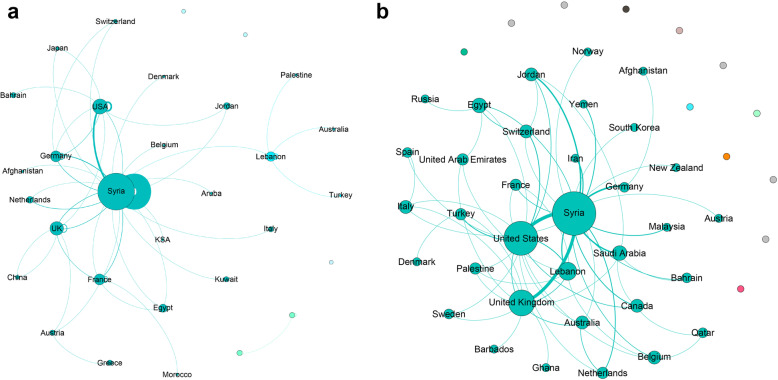
Social network analysis between first and senior authors’ countries of affiliation in health-related articles on Syria. Network on left represents pre-conflict time period. Network on the right represents the in-conflict time-period. Unlabeled nodes represent areas that are not connected to the Syria network

Figure 4 shows the social network analysis in a sub-sample of articles that had more than one author and when the country of affiliation of all authors was reported. For pre-conflict articles (*n* = 228, Fig. 4a) there were 31 nodes (countries) collaborating on Syrian health articles. The five countries with the highest degree of centrality were Syria, United States, United Kingdom, France and Lebanon. During the pre-conflict period, Syrian institutions collaborated 20 times among each other, 8 times with the United States, 7 times with the United Kingdom, 6 times with France, and 6 times with Lebanon. Seven communities (or clusters) of highly interconnected groups were identified and color-coded.

For in-conflict period articles (*n* = 573, Fig. 4b), there were 46 nodes (countries) collaborating on the Syrian health articles. Four of five countries from the pre-conflict period had the highest degree of centrality in-conflict (Syria, United States, United Kingdom, and Lebanon), and the fifth was ranked equal for Canada, Saudi Arabia, and Egypt. During the in-conflict period Syrian institutions collaborated 35 times among each other, 23 times with the United States, 18 times with United Kingdom, 11 times with Lebanon, 8 times with Canada, and 8 times with Saudi Arabia and Egypt respectively. Five communities (or clusters) of highly interconnected groups were identified and color-coded.

Although Canada ranked fifth in degree of centrality in the in-conflict period, it had the highest percentage increase in the number of publications in-conflict (+ 1900%) (Figure S2, Additional file 1). Turkey also ranked third highest in its percentage increase (+ 550%), but was not close to countries with high degree centrality. The highest number of collaborations in-conflict was among institutions from the same countries: Syrian institutions (weight = 238), followed by United States institutions (weight = 61), United Kingdom institutions (weight = 21), Lebanese institutions (weight = 18) and Canadian and Turkish institutions (weight = 10). The highest number of international collaborations was between Syria and the United States (weight = 10), followed by Syria and United Kingdom (weight = 7), and Syria and Germany (weight = 6). An increase in the thickness of the link between Syria and countries like the United States, United Kingdom, Lebanon, Germany, and Saudi Arabia shows an increase in collaboration between authors from those countries and Syrian authors. This is also reflected in the number of publications: pre-conflict, the leading countries of affiliation of first authors were the United States (8.3%), United Kingdom (3.5%), and Lebanon (2.6%) (Tables S5 and S6, Additional file). In-conflict, the United States (12.0%) and the United Kingdom (11.4%) remained in leading positions, followed by Germany (4.5%) and Lebanon (3.9%). Other countries such as France showed a decrease in the number of publications (2.9% vs. 0.7%).

## Discussion

To the best of our knowledge, this is the first study to examine the impact of an armed conflict, past or ongoing, on the health scholarship outputs on Syria. The impact of armed conflict on key aspects of health scholarship on Syria was demonstrated through changes in the number and topic of articles, and international affiliations. There was an increase in news, commentaries and editorial type of publications in articles published in-conflict compared to the pre-conflict period. Although there were more articles published since the onset of the conflict, this was driven by article types other than primary research and was accompanied by an international interest in the conflict that often excluded the involvement of Syrian authors and their institutions.

The proportion of articles with at least one Syrian author was shown to decrease in-conflict compared to pre-conflict. This finding is in line with other studies on countries in the region that experienced a drop in their publications during wartime. For example, both Lebanon and Kuwait witnessed more publications after their respective conflicts in 1992 and 1993 [8]. A bibliometric analysis of research from the Ivory Coast also showed an increase in non-Ivorian first and last authors following periods of political instability and economic deterioration [11], which along with our study adds weight to the hypothesis that increased research productivity may come at the expense of decreased involvement of local institutions. This may also reflect the growing interest in knowledge on internally displaced people and conflict emergencies. However, not all types of conflicts lead to decreased research productivity. For example, the study by Ibrahim (2018) showed that scientific research productivity in Arab countries doubled after the Arab Spring (2006–2010 vs 2011–2015) [9]. The latter study assessed articles conducted by Arab authors as identified by their country of affiliation, in contrast to our review which assessed articles by their content irrespective of the authors’ institutional location. Furthermore, the Arab Spring manifested mostly as protests, making Ibrahim (2018) less applicable to intense, protracted armed conflicts such as that seen in Syria.

When looking at international collaborations broadly, in the case of Syria, the decrease in collaborations with Syrian authors compared to non-Syrian authors in-conflict may be explained by the research sanctions applied on some research centers during the conflict. In line with these finding, Syrian and regional authors have also been marginalized in Syrian refugee research as shown by a recently published article assessing patterns of authorship in health-research related to the Syrian conflict. The findings showed that 92% of articles on Syrian refugees involved non-Syrian affiliations and lacked Syrian institution affiliations, and only 55% of papers included authors from countries neighboring Syria [17]. For the subgroup of articles that included Syrian researchers in our network analysis, more collaboration with some countries was seen in-conflict compared to pre-conflict; for example a higher number of collaborations with countries such as the US, Canada, UK, and Lebanon, appeared in-conflict compared to pre-conflict. Leading countries forming collaborations with Syrian authors were international rather than regional, and when considering the regional countries, Lebanon, Saudi Arabia and Egypt followed other high-income countries’ article collaborations with Syrian authors. Although this may be highly related to research capacity, encouraging regional collaborations is also important.

Our analysis showed a relative increase in mental health and injuries research in the in-conflict period, while there was a decrease in tobacco related topics, and almost no change for other non-communicable diseases. This may reflect donor interests and shows a clear research need for non-communicable disease research in conflict settings. In fact, a recent systematic review showed armed conflict was associated with coronary heart disease, cerebrovascular and endocrine diseases, in addition to increased blood pressure, lipids, alcohol and tobacco use [18]. Therefore, further research on non-communicable disease risk in Syria is needed to inform national public health priorities and policies during conflict. The increase in reporting of no funding in the research type of article suggests that funding is emerging as an important factor for conducting research in conflict. This needs to be explored further to understand the source of reporting, whether institutional or by authors themselves, considering the type of publication. In a survey for research institutions in the EMR, including Syria, limited national funding for health research was reported by the majority of institutions [19]. This is in line with our findings where less than a third of the articles were reported as funded and with no significant changes in-conflict. The increase in ethical approval reporting may be explained by a general improvement in the research governance of institutions and journals over time, confounding the relationship with the Syrian conflict. It can also be explained by the large proportion of the research being led by authors from high income countries, where research systems are more developed and impose additional requirements for conducting research.

Although we conducted a large scoping study following a systematic methodology, nevertheless, this review is not without its limitations. First, we restricted our time period of analysis from 1991 to 2017, and earlier published research may focus on different topics than those we captured. However, based on previous research in this area, we expect the number of articles published before 1991 to be minimal [13]. Second, our analysis only included articles published in journals indexed in seven databases. Articles published in non-indexed local university journals were not included. Third, we focused our review on human and health systems research and excluded medical research on animals and plants. It is possible that armed conflict may affect medical research on animals and plants in a different way and this should be explored in further research. Fourth, we excluded health-related research on Syrian refugees despite increasing international research interest on this population. However, we felt that bibliometric analyses of articles on Syrian refugees required a different analytical approach (e.g. less emphasis on collaborations with Syrian institutions) so this will form the basis of a future review. Fifth, misclassification of articles as pre- and in-conflict is a possibility despite our assumptions that research articles are published with a lag of several years. However, given that 2011 articles consisted only 5.5% of all publications, and research papers for that year were only 3.8%, the effect of misclassification of conflict time period on our analysis may be negligible. Finally, our social network analysis was limited to a sub-sample of articles that contained full information on their countries of affiliation, and we were unable to conduct a missing data analysis to see whether excluded articles were systematically different.

Our findings have a number of research and policy implications. The features of published articles have been shown to change after the start of armed conflict. It is important to be aware of the health needs and priorities of people living in conflict areas so that research is not purely driven by funding and external aid. Scholarship on conflict and health must take note of the political economy of research under such circumstance as has been shown for research on Syrian refugees by UK-funded projects, for example. In these projects, the production of social research on Syrian refugees has been described as being ghost produced by research assistants working overseas in countries hosting refugees such as Lebanon [20]. However, the focus of the latter research is firmly on Syrian refugees, and little is known about institutions and researchers who remained in Syria. This raises questions as to whether international research on Syria is involving and adequately acknowledging Syrian researchers through authorship. Additional studies on the types of institutions involved in health research can help characterize research actors and reveal any academic, humanitarian, and policy research partnerships taking place among different collaborators. Key informant interviews may also explore the ways in which conflict affects ethics, funding and ownership of research publications. In terms of topics, focusing on certain areas may reflect a neglect of other priority health topics, such as non-communicable diseases, which in the long run will be unmasked and may require funding and action.

## Conclusion

The Syrian conflict was associated with a change in the rates, types, and topics of the health-related articles, and authors’ affiliations. Our findings show a decreased involvement of local authors compared to non-Syrian authors and thus advocate for the importance of including local scholars when publishing journal articles. When considering collaborations in conflict, less regional and more international collaborations took place after the start of conflict. Our findings also have implications for the development of inclusive research collaborations, the prioritization of research funding, and the promotion of the ethics of conducting research in complex humanitarian settings.

## Supplementary Information

**Additional file 1:** **Table S1:** full search strategy. **Table S2:** Top 15 journals publishing on Syria before and after the start of conflict. **Table S3:** Funding for research papers. **Table S4:** List of funding institutions for all funded publications. **Table S5:** Top publishing countries of affiliation for first author for articles on Syria (with more than 15 articles). **Table S6:** Top countries of affiliation of last author in publications with more than one author. **Figure S1:** Publications including any Syria affiliated author vs. non-Syrian affiliations over year of publication. **Figure S2:** percentage change pre- and post-conflict in publications for countries of affiliation with highest number of publications on Syria.

## Data Availability

The datasets used and/or analysed during the current study are available from the corresponding author on reasonable request.
