# Supplementary material for: Health-related articles on Syria before and after the start of armed conflict: a scoping review for The Lancet-American University of Beirut Commission on Syria
Source: Confl Health. 2020 Nov 5;14:73. doi: 10.1186/s13031-020-00316-7 (PMC7643257; doi:10.1186/s13031-020-00316-7)

**Additional file 1 to: The health literature on Syria before and after the start of armed conflict: A scoping review for *The* *Lancet*-American University of Beirut Commission on Syria**

**TABLES**

Search strategy

Table S1: Search terms – search run on 14th April 2017

| Search numbers | Search terms | Embase, Global Health, Medline, PsychInfo | Web of Science | PubMed | Scopus |
| --- | --- | --- | --- | --- | --- |
| 1 | Syria/exp or Syria or Syrian or Syrias or Syrians | 33,125 | 11,837 | - | - |
| 2 | hamster* | 183,812 | 60,042 | - | - |
| 3 | 1 not 2 | 6,052 | 4,480 | 4,317 | 7,070 |
| Total after merging and removing duplicates | 13,758 | | | | |

Note: search limited to 1991-present

Scopus: Limited to medicine, psychology, dentistry, health professions, and neuroscience databases

Web of Science: All social science databases removed

Embase, Global Health, Medline, PsychInfo (combined into one)

1. syrian arab republic/exp OR syria/exp OR syria OR syrian OR syrias OR syrians

2. hamster*

3. 1 NOT 2

Scopus

ALL ( syria OR syrian OR syrias OR syrians ) AND PUBYEAR > 1990 AND NOT hamster* AND ( LIMIT-TO ( SUBJAREA , "MEDI" ) OR LIMIT-TO ( SUBJAREA , "PSYC" ) OR LIMIT-TO ( SUBJAREA , "DENT" ) OR LIMIT-TO ( SUBJAREA , "NURS" ) OR LIMIT-TO ( SUBJAREA , "HEAL" ) OR LIMIT-TO ( SUBJAREA , "NEUR" ) OR LIMIT-TO ( SUBJAREA , "Undefined" ) ) AND ( LIMIT-TO ( SRCTYPE , "Undefined" ) )

Web of Science (removed all Social Science Databases)

1. TS=(‘Syrian Arab Republic’ or Syria or Syrias or Syrian or Syrians’)

2. TS=hamster*

3. #1 not #2

PubMed

(((syria or syrian or syrians or syrians)) NOT hamster*) AND ("1991"[Date - Publication] : "3000"[Date - Publication])

Table S2 Top 15 journals publishing on Syria before and after the start of conflict

| **Pre-conflict (Inside Syria)** | **N (%)** | **In-conflict (Inside Syria)** | **N (%)** |
| --- | --- | --- | --- |
| Saudi Medical Journal | 22(7.0) | The Lancet | 65(7.8) |
| Eastern Mediterranean Health Journal | 20(6.4) | BMJ | 33(4.0) |
| Saudi Journal of Kidney Diseases and Transplantation | 9(2.8) | Eastern Mediterranean Health Journal | 21(2.5) |
| International Journal of Tuberculosis | 8(2.5) | Avicenna journal of medicine | 18(2.1) |
| Asian Cardiovascular and Thoracic Annals | 5(1.6) | International Journal of Pharmaceutical | 13(1.5) |
| Dermatology Online Journal | 5(1.6) | BMJ (Online) | 11(1.3) |
| BMC Public Health | 4(1.2) | CMAJ | 8 (0.9) |
| The Lancet | 4(1.2) | Special issue: Programming experiences | 8(0.9) |

Table S3 Funding for research papers, n%

|  | | Total  N=809  n (%) | Pre-conflict  (N=268)  n (%) | In-conflict  (N=541)  n (%) | p-value |
| --- | --- | --- | --- | --- | --- |
| Funding | Funded | 220 (27.1) | 80 (29.8) | 140 (25.8) | p-value=0.000 |
|  | Reported as not funded | 82 (10.1) | 3 (1.1) | 79 (14.6) |  |
|  | Not reported | 507 (62.6) | 185 (36.4) | 322 (63.5) |  |

Fisher’s exact test; p<0.05 statistically significant; research papers included primary research, secondary research and conference proceedings.

Table S4 List of funding institutions for all funded publications, n%

| Name of Funding Body | N (%)  (N=231) | Pre-conflict  (N=82) | In-conflict  (N=149) |
| --- | --- | --- | --- |
| Damascus University | 28 (12.1) | 24 (2.9) | 4 (2.4) |
| United States Public Health Service | 27 (11.6) | 5 (0.6) | 22 (7.0) |
| Atomic Energy Commission of Syria | 24 (10.3) | 0 (0) | 24 (2.9) |
| World Health Organization | 9 (3.9) | 2 (0.2) | 7 (2.2) |
| University of Aleppo | 8 (3.4) | 0 (0) | 8 (0.9) |
| National Institute on Drug Abuse | 8 (3.4) | 3 (3.6) | 5 (3.3) |
| Canadian Institute for Health research | 7 (3.0) | 2 (0.6) | 5 (0.6) |
| American University of Beirut | 7 (3.0) | 5 (6.1) | 2 (1.3) |
| Bill & Melinda Gates Foundation | 4 (1.7) | 0 (0) | 4 (2.6) |

*Funding body chosen for analysis is the first body that is reported in an article when there is more than 1 funding body, p-value=0.000

Table S5 Top publishing countries of affiliation for first author for articles on Syria (with more than 15 articles)

|  | | Total  N = 1138 (%) | Pre-conflict  (N=312) | In-conflict  (N=826) | p-value |
| --- | --- | --- | --- | --- | --- |
| Country | Syria | 534 (46.9) | 192 (61.5) | 342 (41.4) | <0.001 |
|  | United States | 125 (10.9) | 26 (8.3) | 99 (12.0) |  |
|  | United Kingdom | 105 (9.2) | 11 (3.5) | 94 (11.4) |  |
|  | Lebanon | 40 (3.5) | 8 (2.5) | 32 (3.9) |  |
|  | Germany | 25 (2.2) | 14 (1.3) | 11 (4.5) |  |
|  | Canada | 21 (1.8) | 1 (0.3) | 20 (2.4) |  |
|  | Jordan | 16 (1.4) | 4 (1.2) | 12 (1.4) |  |
|  | Egypt | 15 (1.3) | 6 (1.9) | 9 (1.0) |  |
|  | France | 15 (1.3) | 9 (2.9) | 6 (0.7) |  |
|  | Turkey | 15 (1.3) | 2 (0.6) | 13 (1.5) |  |

Chi2 test; p<0.05 statistically significant

Table S6 Top countries of affiliation of last author in publications with more than one author

|  |  | Total  N=808 (%) | Pre-conflict  (N=236) | In-conflict  (N=572) | p-value* |
| --- | --- | --- | --- | --- | --- |
| Country | Syria | 402 (49.7) | 127 (53.8) | 275 (48.0) | 0.190 |
|  | United States | 104 (12.8) | 28 (11.8) | 76 (13.2) |  |
|  | United Kingdom | 51 (6.3) | 12 (5.0) | 39 (6.82) |  |
|  | Lebanon | 33 (4.0) | 8 (3.3) | 25 (4.37) |  |
|  | Germany | 26 (3.2) | 16 (2.8) | 10 (4.24) |  |
|  | France | 16 (1.9) | 8 (3.3) | 8 (1.40) |  |
|  | Canada | 15 (1.8) | 2 (0.8) | 13 (2.27) |  |
|  | Saudi Arabia | 12 (1.4) | 1 (0.4) | 11 (1.92) |  |

Chi2 test; p<0.05 statistically significant

**FIGURES**

Figure S1 Publications including any Syria affiliated author vs. non-Syrian affiliations over year of publication


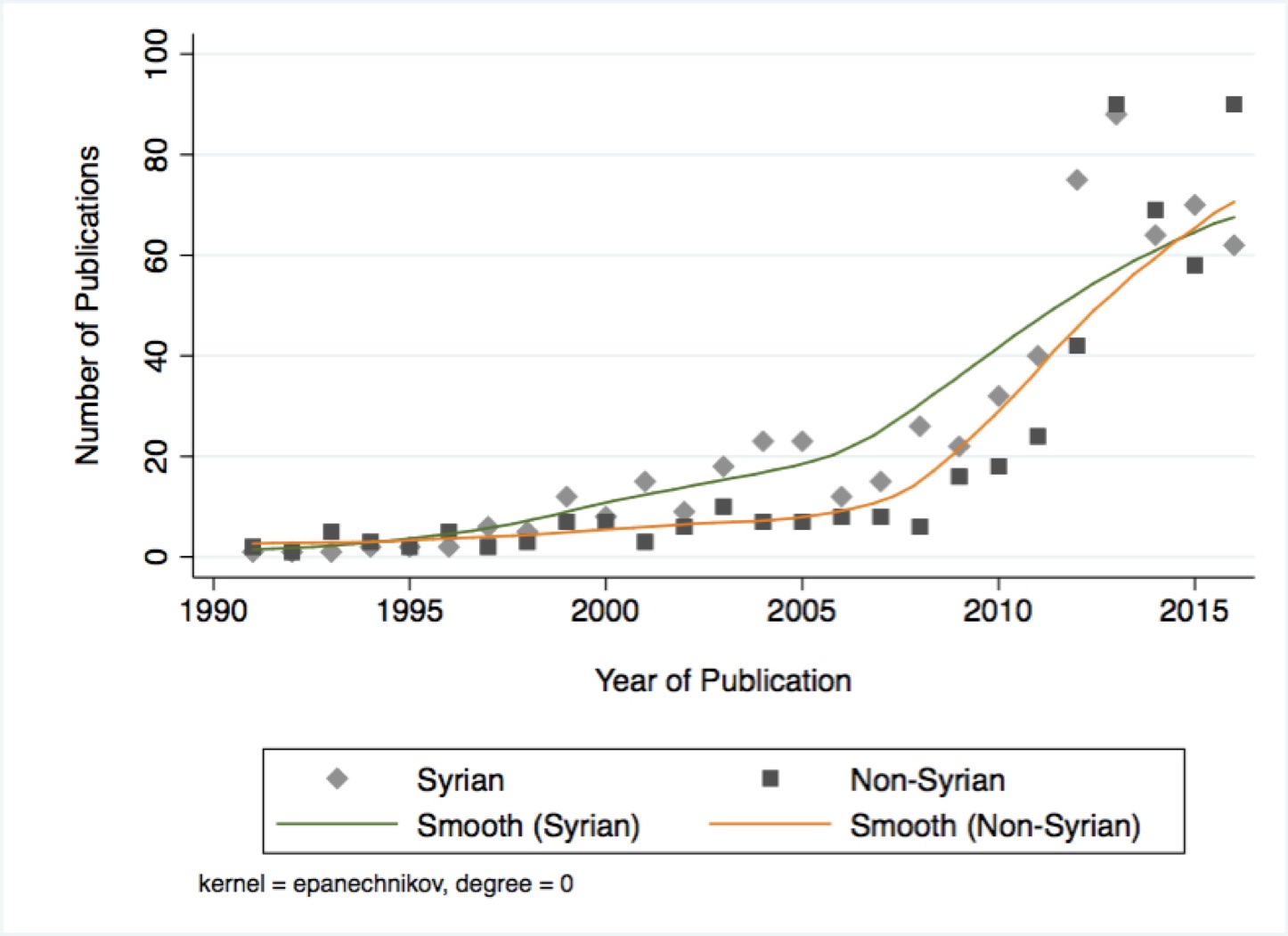


Figure S2


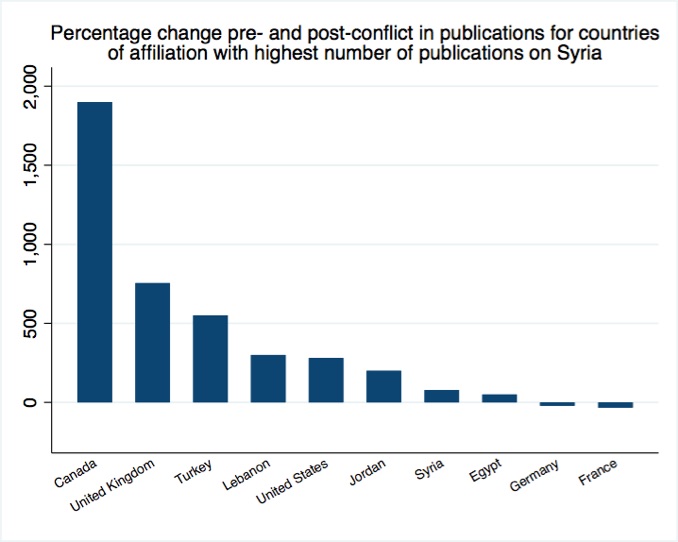

Supplement: Supplementary file 1 — Additional file 1: Table S1: full search strategy. Table S2: Top 15 journals publishing on Syria before and after the start of conflict. Table S3: Funding for research papers. Table S4: List of funding institutions for all funded publications. Table S5: Top publishing countries of affiliation for first author for articles on Syria (with more than 15 articles). Table S6: Top countries of affiliation of last author in publications with more than one author. Figure S1: Publications including any Syria affiliated author vs. non-Syrian affiliations over year of publication. Figure S2: percentage change pre- and post-conflict in publications for countries of affiliation with highest number of publications on Syria. [file 13031_2020_316_MOESM1_ESM.docx]
